# Prevalence and risk factors for allergic sensitization: 3 cross-sectional studies among schoolchildren from 1996 to 2017

**DOI:** 10.1016/j.jacig.2023.100150

**Published:** 2023-07-19

**Authors:** Eva Rönmark, Joakim Bunne, Anders Bjerg, Matthew Perzanowski, Anna Winberg, Martin Andersson, Thomas Platts-Mills, Linnea Hedman

**Affiliations:** a Division of Sustainable Health, Department of Public Health and Clinical Medicine, The OLIN Unit, Umeå University, Umeå; b Department of Women’s and Children’s Health, Karolinska Institute, Stockholm; c Department of Environmental Health Sciences, Mailman School of Public Health, Columbia University, New York; d Department of Clinical Sciences, Pediatrics Unit, Umeå University, Umeå; e Division of Allergy & Clinical Immunology, University of Virginia, Charlottesville

**Keywords:** Allergic sensitization, asthma, allergic rhinitis, epidemiology, OLIN, schoolchildren, skin prick test

## Abstract

**Background:**

The prevalence of allergic sensitization and allergic diseases has increased for decades in Northern Europe, but recent studies are lacking.

**Objective:**

We sought to study the prevalence trends of allergic sensitization, associated risk factors, and the association with asthma and allergic rhinitis (AR) among children in Northern Sweden.

**Methods:**

Three cohorts of children aged 7 to 8 years participated in a skin prick test (SPT) with 10 airborne allergens in 1996, 2006, and 2017, with 2148, 1693, and 1762 participants tested, respectively, representing 87% to 90% of schoolchildren in the catchment communities. Adjusted Poisson regression was used to identify risk factors for allergic sensitization and the association with asthma and AR.

**Results:**

The prevalence of any positive SPT response increased from 21% in 1996 to 30% in 2006 and remained at 30% in 2017 (*P* < .001). Sensitization to cat was the most common for all the years. The risk factor pattern for a positive SPT response was similar in all examinations, with positive and significant associations with a family history of allergy (risk ratio, 1.4-1.5) and negative and significant associations with having a cat at home (risk ratio, 0.7-0.8). The prevalence of physician-diagnosed asthma increased, but the association with allergic sensitization weakened. The opposite trends were found for AR—decreasing prevalence and strengthened association with allergic sensitization.

**Conclusions:**

The prevalence of allergic sensitization increased from 1996 to 2006 but plateaued in the next decade, whereas the risk factor pattern remained stable. The diverging trends of associations between allergic sensitization and asthma and AR suggest secular trends in the clinical management of allergic diseases.

Allergic diseases have been a growing global health concern for decades.^1^ However, recent global trends in the prevalence of asthma and asthma symptoms show diverging results. In some areas, the prevalence of asthma is still increasing, whereas in others, the trend has plateaued.^2^ A similar development has been reported for allergic rhinitis (AR).^3^ Allergic sensitization is a key step in the development of allergic diseases, and sensitization to airborne allergens is consistently the strongest identified risk factor for asthma and AR.^4^, ^5^, ^6^ Even so, few large-scale studies about trends in allergic sensitization have been published, and there are fewer that have a high-enough participation in a studied community to truly capture a population prevalence. Some of them report no further increase, although, as for studies in asthma and AR, the results diverge.^7^, ^8^, ^9^, ^10^, ^11^, ^12^, ^13^ Studies reflecting the recent trends are lacking.

The prevalence of allergic sensitization to airborne allergens increases by age, because of high incidence and a high level of persistence from childhood to adulthood.^14^^,^^15^ Thus, the prevalence of allergic sensitization among children has a major effect on the prevalence among adults, and subsequently on the incidence of asthma and AR.^16^ Although dust mites are a major sensitizer in warmer climates,^11^^,^^14^^,^^17^ in colder and drier areas, pollen and furry animals are the dominating airborne sensitizers.^6^^,^^12^^,^^18^

A family history of allergy is a well-established risk factor for allergic sensitization,^12^^,^^19^ but less is known about other risk factors. It is suggested that exposure to a diverse microbiome, particularly in early life, may stimulate the immune system and decrease the risk of developing allergic sensitization.^20^, ^21^, ^22^ Among such exposures, for which a negative association with sensitization has been shown, are rural living, living on a farm, having many siblings, and having pets at home.^20^, ^21^, ^22^, ^23^, ^24^, ^25^, ^26^, ^27^, ^28^ Even though the complex mechanism of tolerance development is not yet fully understood, it has been shown that the controlling effect by having a cat at home includes development of blocking IgG antibodies to Fel d 1.^25^^,^^29^ One factor contributing to the scarceness of studies assessing the prevalence trends of allergic sensitization in the general population is the need for objective testing of allergic sensitization. Skin prick test (SPT) for airborne allergens is a valid and useful method for large-scale epidemiological studies.^30^^,^^31^

A goal of the Obstructive Lung Disease in Northern Sweden studies was to measure the prevalence of allergic sensitization, asthma, and AR. From these studies, we previously reported a significant increase (from 21% to 30%) in allergic sensitization to airborne allergens among 8-year-old schoolchildren from 1996 to 2006.^12^ The magnitude of difference between the cohorts remained at age 12 years when they were tested again.^31^ We have since recruited a third cohort of the same age in the same area, which was examined by identical methods. The aim of the present study was to investigate time trends of allergic sensitization to airborne allergens, its risk factors, and the association with asthma and AR on the basis of 3 cross-sectional studies of 8-year-old schoolchildren investigated in 1996, 2006, and 2017, respectively.

## Methods

### Study population

The present study includes 3 cohorts within the Obstructive Lung Disease in Northern Sweden studies. In 1996,^4^ 2006,^12^ and 2017, all children in the first and second grades (median age, 8 years) in 2 municipalities in Northern Sweden were invited to participate in an SPT and a parental questionnaire study. In total, 2148 (88%) participated in both the questionnaire study and the SPT in 1996, 1693 (89%) in 2006, and 1762 (87%) in 2017. Written informed consent was obtained from the parents. The Regional Ethical Committee at Umeå University approved the studies (Dnr 96-032, Dnr 05-157M, and Dnr 2016/454-31).

### Questionnaire

The questionnaire included the International Study of Allergy and Asthma in Children questionnaire,^32^ with additional questions about possible risk factors including family history of allergic diseases, number of siblings and birth order, birth weight, length of breast-feeding, respiratory infections, past or present cat or dog at home, parental smoking, house dampness, and other living conditions.^12^ The questionnaire was distributed by the schoolteachers and completed by the parents at home. The definitions of asthma, AR, and risk factors were based on the questionnaire and are presented in Table E1 (in the Online Repository available at www.jaci-global.org).

### Skin prick tests

In all 3 cohorts, the SPT was performed in the schools by a small group of specially trained research nurses, and all tests were performed during the same period (February to April). The methods followed the European Academy of Allergology and Clinical Immunology recommendations.^33^ Ten common airborne allergens were used: birch, timothy, mugwort, dog, cat, horse, *Dermatophagoides farinae*, *Dermatophagoides pteronyssinus*, Cladosporium, and Alternaria (Soluprick, ALK, Hørsholm, Denmark)*.* The potency of the extracts was 10 histamine equivalent prick, except the 2 molds, which were 1:20 wt/vol. Histamine (10 mg/mL) and glycerol were used as positive and negative controls, respectively. A positive reaction was recorded if the mean wheal was greater than or equal to 3 mm after 15 minutes. Allergic sensitization was defined as a positive SPT response to any airborne allergens tested. The SPTs in the first and second cohorts have been validated against specific IgE and showed high agreement.^12^^,^^31^

### Analyses

IBM SPSS Statistics version 28.0 (Armonk, NY) was used for statistical analyses. The χ^2^ test was used for comparison of proportions or the ANOVA for comparison of mean values between groups. A *P* value less than .05 was considered statistically significant. Poisson regression was used to identify risk factors in each survey expressed as risk ratio (RR) with 95% CI. Independent variables significantly associated with allergic sensitization in unadjusted analyses in any survey were included in the adjusted analyses. In a joint analysis of all 3 surveys, adjusted for study year and potential confounders, the change in prevalence from 1996 to 2006 and 2017, respectively, was explored and expressed as RR (95% CI). In the same analysis, variables suggestive of exposure to a more diverse microbiome were aggregated to construct a compound variable. This variable counted the number of positive answers to 4 questionnaire items: rural living during first year of life, ever living at a farm, ever having a cat at home, and ever having a dog at home, which were grouped as 0, 1, 2, and 3 to 4 exposures. The association between allergic sensitization and asthma and AR, respectively, in each survey was also analyzed by Poisson regression analyses, adjusted for known risk factors in the cohorts.^4^^,^^12^^,^^25^^,^^31^ The population-attributable risk (PAR) of allergic sensitization for asthma and AR was calculated for each year by the formula (*P*_p_ − *P*_un_)/*P*_p_, where *P*_p_ is the prevalence in the whole population and *P*_un_ is the prevalence among the nonsensitized.

## Results

### Prevalence of allergic sensitization

**Fig 1:**
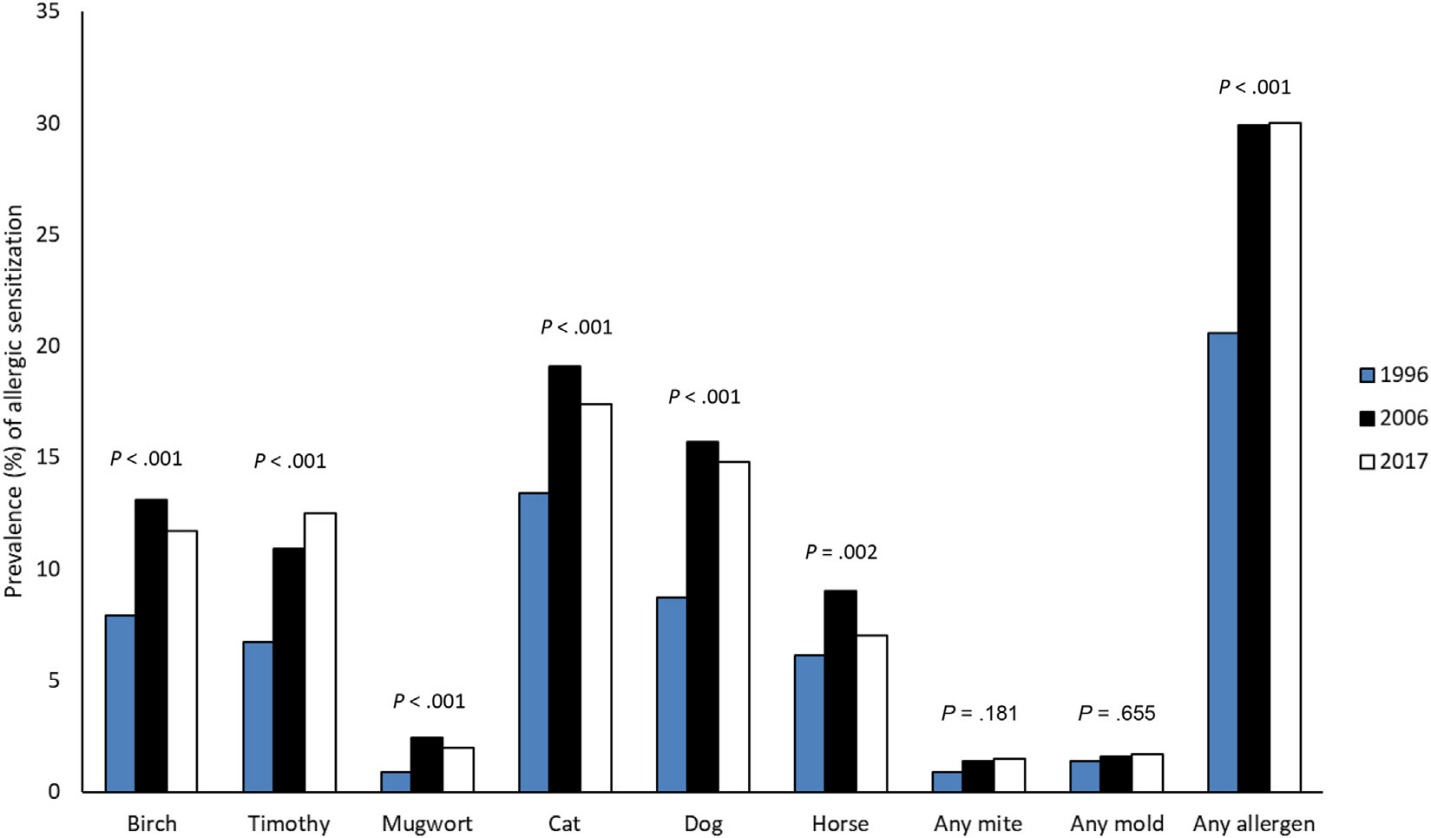
Prevalence (%) of allergic sensitization to airborne allergens defined by SPT among 8-year-old children by study year. Difference in proportions was analyzed using the χ^2^ test.

An increase in prevalence of any positive SPT response during the study period was observed (*P* < .001) (from 20.6% in 1996 to 29.9% in 2006), whereas it remained almost identical (at 30.0%) in 2017. The pattern of allergic sensitization to the tested airborne allergens was similar in all surveys, with sensitization to cat and dog being the most common and mite and mold the least common sensitizers (Fig 1 and Table I). Overall, allergic sensitization was more common among boys, although not statistically significantly for all allergens (Table I). In an adjusted analysis with year 1996 as reference, the RR for any positive SPT response was 1.46 (95% CI, 1.28-1.66) in 2006 and 1.44 (95% CI, 1.27-1.64) in 2017 (data not in table). Sensitivity analyses based on larger cutoffs for definition of a positive response showed lower prevalence; however, the pattern in prevalence trends remained (see Table E2 in this article’s Online Repository at www.jaci-global.org).

**Table I:** Prevalence (%) of allergic sensitization to airborne allergens among 8-year-old schoolchildren, by year of examination and sex∗

Allergen	1996 (n = 2148)			2006 (n = 1693)			2017 (n = 1762)			
	Girls	Boys	Difference by sex, *P* value	Girls	Boys	Difference by sex, *P* value	Girls	Boys	Difference by sex, *P* value	Difference by year, *P* value
Birch	6.7	9.2	.036	11.9	14.2	.170	9.6	14.9	<.001	<.001
Timothy	5.2	8.1	.007	8.7	13.1	.004	11.0	12.3	.384	<.001
Mugwort	0.8	1.0	.649	2.6	2.2	.592	2.2	1.7	.465	.001
Any pollen	10.3	14.4	.004	16.7	19.8	.098	17.4	20.4	.108	<.001
Cat	12.2	14.6	.107	17.7	20.5	.139	14.8	19.5	.009	<.001
Dog	8.1	9.3	.343	12.9	18.5	.001	12.3	17.2	.004	<.001
Horse	4.8	7.4	.011	8.0	10.0	.159	5.6	8.6	.012	.002
Any animal	15.5	17.1	.306	20.5	25.8	.011	19.1	24.2	.008	<.001
*D pteronyssinus*	0.2	0.8	.033	0.8	1.9	.066	1.3	0.9	.408	.021
*D farina*	0	1.3	<.001	0.4	1.3	.035	1.1	0.5	.903	.413
Any mite	0.2	1.7	<.001	0.8	2.0	.045	1.6	1.4	.721	.181
Cladosporium	0.7	1.5	.096	0.6	2.0	.011	1.1	1.3	.594	.809
Alternaria	0.3	0.9	.078	0.5	0.9	.259	1.2	0.9	.555	.223
Any mold	0.8	1.9	.037	0.8	2.4	.013	1.8	1.6	.737	.655
Any allergen	19.0	22.3	.059	27.2	32.6	.016	27.6	32.1	.040	<.001

∗ Differences in proportion were analyzed using the χ^2^ test.

Among sensitized children, the mean number of positive SPT reactions was similar in all surveys, although somewhat higher in 2006 (2.49) than in 1996 (2.23) and 2017 (2.31) (*P* = .020). The mean size of the reactions was similar in 1996, 2006, and 2017 (see Table E3 in this article’s Online Repository at www.jaci-global.org).

### Risk factors for allergic sensitization

The prevalence of potential risk factors is provided in Table II. The proportion with a family history of allergy, living in a house the first year of life, and ever having a dog at home increased significantly in each subsequent survey, whereas having 2 or 3 or more siblings, having had any severe respiratory infection, and maternal smoking decreased significantly over the period of the study. Other factors remained unchanged.

**Table II:** Prevalence (%) of risk factors and the association (RR) with allergic sensitization defined as any positive SPT response to airborne allergens, by year of examination

Risk factor	Prevalence of risk factor (%), by year of survey			*P* value	RR (95% CI)∗ for any positive SPT response, by year of survey		
	1996	2006	2017		1996	2006	2017
The male sex	50.0	50.4	51.9	.467	1.17 (0.97-1.42)	**1.21 (1.01-1.44)**	1.16 (0.98-1.38)
Family history allergy	37.0	42.1	45.2	<.001	**1.47 (1.22-1.77)**	**1.47 (1.24-1.76)**	**1.53 (1.29-1.81)**
No. of siblings							
0	6.1	7.1	7.8	<.001	1	1	1
1	42.2	50.2	47.8		0.79 (0.55-1.13)	0.97 (0.68-1.37)	0.85 (0.63-1.16)
2	32.8	25.2	25.4		0.73 (0.51-1.06)	1.07 (0.74-1.54)	0.74 (0.53-1.04)
≥3	18.8	17.5	19.1		**0.66 (0.44-0.97)**	0.90 (0.61-1.33)	0.82 (0.58-1.51)
Type of living first year of life							
House	49.8	55.8	58.7	<.001	1	1	1
Apartment	50.2	44.2	41.3		**1.23 (1.02-1.49)**	1.18 (0.99-1.41)	1.07 (0.90-1.28)
Ever having a cat at home	26.5	24.4	24.0	.153	**0.65 (0.51-0.82)**	**0.72 (0.57-0.90)**	**0.76 (0.61-0.95)**
Ever having a dog at home	33.8	36.1	37.9	.029	**0.74 (0.60-0.91)**	**0.74 (0.61-0.90)**	**0.82 (0.68-0.98)**
Furry animal at home first 2 y of life	34.4	35.6	38.3	.043	**0.81 (0.66-0.99)**	**0.78 (0.66-0.97)**	**0.80 (0.67-0.96)**
Rural living in first year of life	26.6	28.2	26.1	.010	**0.79 (0.63-0.99)**	0.98 (0.82-1.22)	1.09 (0.89-1.32)
Ever living on a farm	2.7	2.5	3.3	.362	**0.33 (0.12-0.89)**	0.71 (0.37-1.38)	0.98 (0.62-1.62)
Birthweight <2500 g	3.7	3.7	4.6	.300	0.96 (0.57-1.60)	1.05 (0.67-1.67)	1.18 (0.80-1.74)
Breast-feeding <3 mo	14.6	9.5	14.7	<.001	0.90 (0.68-1.19)	1.05 (0.78-1.41)	0.97 (0.78-1.27)
Severe respiratory infection	58.3	27.8	22.7	<.001	1.00 (0.83-1.22)	1.04 (0.85-1.26)	1.06 (0.87-1.30)
Maternal smoking	31.8	15.5	7.0	<.001	0.88 (0.71-1.08)	0.95 (0.74-1.22)	0.96 (0.68-1.35)
Mother smoked during pregnancy	25.4	11.0	3.2	<.001	0.83 (0.67-1.04)	0.86 (0.64-1.16)	0.84 (0.49-1.42)
Dampness at home	20.8	12.3	15.6	<.001	1.01 (0.18-1.28)	0.98 (0.75-1.29)	0.97 (0.76-1.23)
Heavy traffic road close to home	44.1	42.2	43.0	.486	1.07 (0.89-1.30)	1.18 (0.99-1.41)	1.05 (0.88-1.25)

∗ Unadjusted Poisson regression analyses. Significant associations are indicated in boldface.

**Fig 2:**
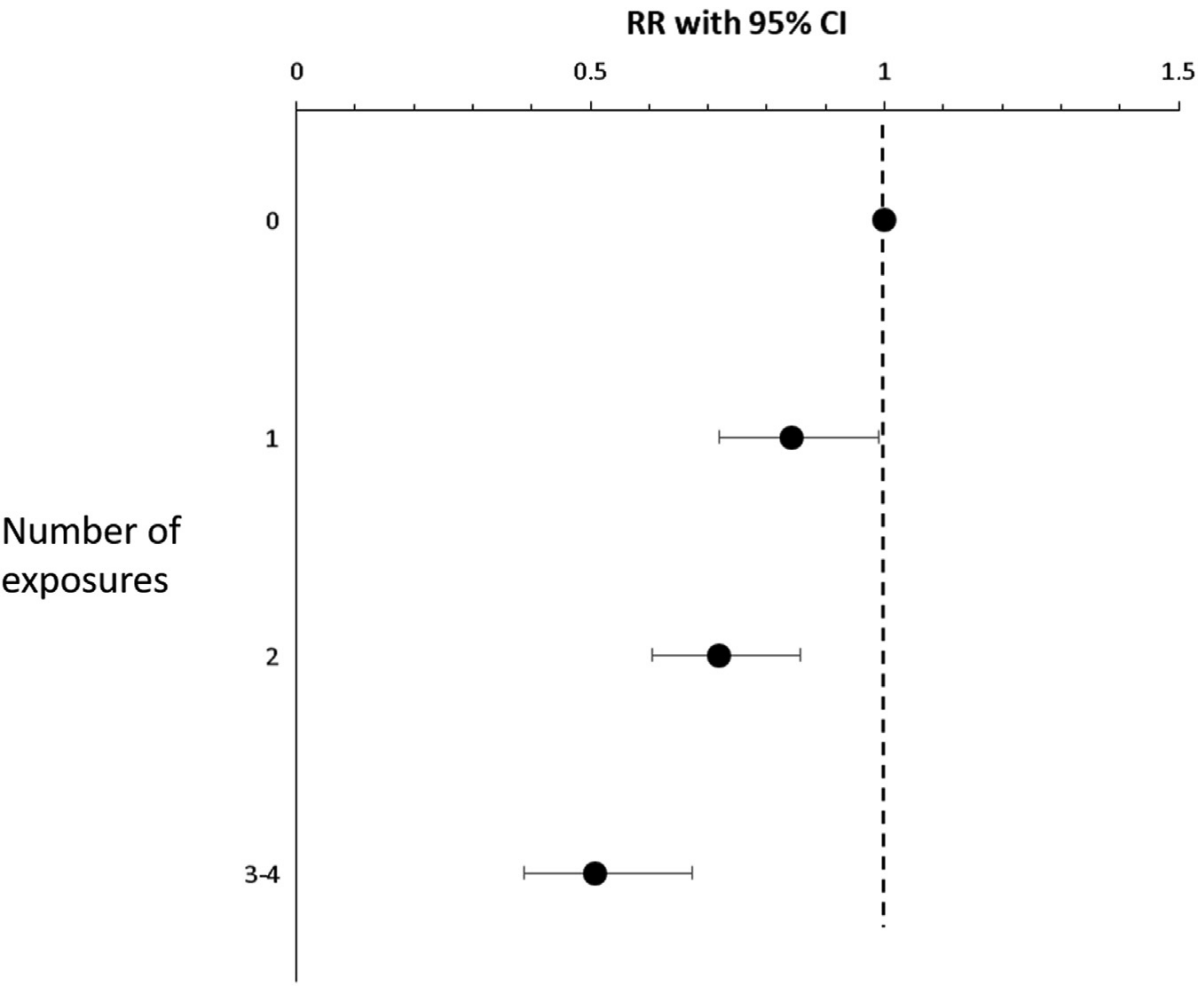
Risk for allergic sensitization in relation to number of exposures suggestive of higher microbiome diversity (ever having a dog at home, ever having a cat at home, rural living first year of life, and ever living on a farm) in a pooled analysis of all 3 cohorts. Analyzed by Poisson regression and adjusted for sex, family history of allergic disease, number of siblings, type of living first year of life, and study year, expressed as RR with 95% CI.

Factors that were significantly associated with any positive SPT reaction in unadjusted analyses in any of the 3 surveys are presented in Table II. A family history of allergy was associated with an increased risk for any positive SPT reaction in all surveys (RR, 1.47-1.53), whereas the variables ever having a cat or a dog at home as well as having a furry animal at home in the first 2 years of life were consistently associated with a decreased risk (RR, 0.65-0.82). Having 3 or more siblings, rural living, and living on a farm were negatively associated with sensitization in 1996 but not in 2006 or in 2017. In the adjusted analyses (Table III) including all variables in the table (model I), a family history of allergy remained a significant risk factor in all surveys, and the male sex was significant in 2006 but not in 1996 or in 2017. Ever having a cat at home was significantly associated with a decreased risk in 1996 (RR, 0.69 [95% CI, 053.-0.91]). To address a strong covariance between the variables ever having a cat at home, ever having a dog at home, furry animal first year of life, rural living first year of life, and ever living at a farm, these variables were also included one by one (model II). In this model, ever having a cat at home was a statistically significant negative predictor for allergic sensitization in all surveys (RR, 0.70-0.78) and the pattern was similar for ever having a dog at home, although significantly so only in 2006. Other variables adjusted for remained unchanged (Table III). In a pooled and adjusted analysis of all 3 cohorts, a significant dose-response effect by number of variables suggestive of higher microbiome diversity was found (Fig 2).

**Table III:** Risk factors for allergic sensitization to airborne allergens by study year, analyzed by adjusted Poisson regression models and expressed as RR with 95% CI

	RR (95% CI) for any positive SPT response to any airborne allergen					
	Model I∗			Model II†		
**Risk factor**	**1996**	**2006**	**2017**	**1996**	**2006**	**2017**
Sex, male	1.13 (0.93-1.37)	**1.22 (1.01-1.47)**	1.15 (0.96-1.37)			
Family history allergy	**1.39 (1.14-1.69)**	**1.44 (1.19-1.73)**	**1.49 (1.25-1.79)**			
No. of siblings						
0	1	1	1			
1	0.76 (0.52-1.11)	0.90 (0.62-1.30)	0.83 (0.60-1.14)			
2	0.70 (0.47-1.04)	1.01 (0.69-1.49)	0.75 (0.53-1.05)			
≥3	0.70 (0.46-1.06)	0.86 (0.62-1.30)	0.79 (0.55-1.13)			
Type of living first year of life						
House	1	1	1			
Apartment	1.17 (0.95-1.46)	**1.23 (1.01-1.51)**	1.10 (0.89-1.35)			
Ever having a cat at home	**0.69 (0.53-0.91)**	0.78 (0.61-1.00)	0.80 (0.62-1.03)	**0.70 (0.54-0.89)**	**0.77 (0.61-0.96)**	**0.78 (0.62-0.97)**
Ever having a dog at home	0.78 (0.60-1.03)	0.79 (0.62-1.01)	0.84 (0.65-1.09)	0.80 (0.65-1.00)	**0.78 (0.64-0.95)**	0.83 (0.69-1.00)
Furry animal at home first 2 y of life	1.19 (0.90-1.58)	1.05 (0.81-1.36)	0.97 (0.74-1.27)	0.88 (0.71-1.03)	0.86 (0.71-1.04)	0.83 (0.69-1.00)
Rural living first year of life	1.08 (0.83-1.40)	0.84 (0.67-1.06)	0.82 (0.66-1.03)	1.16 (0.90-1.50)	0.89 (0.71-1.12)	0.88 (0.71-1.10)
Ever living on a farm	0.43 (0.16-1.15)	0.92 (0.45-1.1.87)	1.08 (0.65-1.79)	0.39 (0.15-1.04)	0.81 (0.42-1.58)	1.04 (0.64-1.69)

Data in boldface indicate significant associations.

∗ Model I: All variables presented in the table were included in the model.

† Model II: The presented variables were included one at a time in the model and adjusted for sex, family history of allergy, number of siblings, and type of living first year of life. The associations for these variables remained almost identical in all analyses and as in model I.

### Prevalence of asthma and AR and their association with allergic sensitization

**Fig 3:**
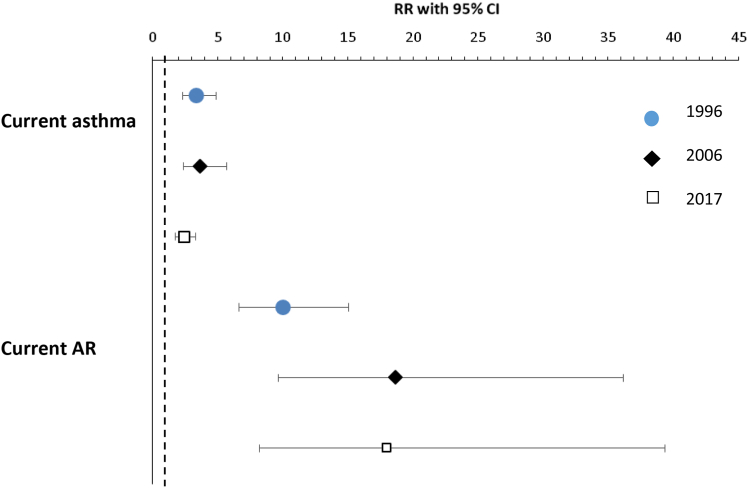
The association between allergic sensitization and current asthma and AR, respectively, by study year. Analyzed by Poisson regression and adjusted for sex, family history of asthma or AR, respectively, maternal smoking in pregnancy, severe respiratory infection, breast-feeding for less than 3 months, and ever having a cat at home, expressed as RR with 95% CI.

The prevalence of current asthma was 5.9% in 1996 and 5.7% in 2006 and increased to 9.2% in 2017 (*P* < .001), whereas the prevalence of wheeze in the last 12 months remained stable at about 11% (Table IV). The pattern was similar among sensitized and nonsensitized children. Asthma medication use increased significantly among nonsensitized children but not among sensitized children. In contrast, the prevalence of current AR decreased significantly from 6.4% in 1996 to 4.0% in 2017 (*P* = .004), which occurred in sensitized and nonsensitized children alike. Symptoms of AR, however, remained stable at about 15% (Table IV). The highly different prevalence trends in allergic sensitization, asthma, and AR, respectively (Table IV), resulted in changes in the strength of associations. Over time, the association between airborne allergen sensitization and current asthma tended to weaken, whereas its association with current AR became stronger (Fig 3). Meanwhile, the associations with the symptom variables remained stable. The PAR of allergic sensitization for physician-diagnosed asthma decreased from 35.9% in 1996 to 26.4% in 2017, whereas for physician-diagnosed AR, the PAR increased from 68.5% in 1996 to 79.6% in 2017.

**Table IV:** Prevalence (%) of asthma and AR by study year and among sensitized and nonsensitized children, respectively

Symptoms and conditions	Among all children				Among sensitized children				Among nonsensitized children			
	1996	2006	2017	Difference by year,	1996	2006	2017	Difference by year,	1996	2006	2017	Difference by year,
	(n = 2148)	(n = 1693)	(n = 1762)	*P* value	(n = 443)	(n = 506)	(n = 528)	*P* value	(n = 1705)	(n = 1187)	(n = 1234)	*P* value
Wheeze last 12 mo	11.3	10.8	11.1	.876	22.6	20.9	19.1	.417	8.3	6.4	7.7	.155
Asthma medication last 12 mo	7.9	8.4	11.9	<.001	18.3	17.6	20.1	.572	5.2	4.5	8.3	<.001
Physician-diagnosed asthma	6.4	7.2	12.5	<.001	15.3	13.6	20.5	.009	4.1	4.5	9.2	<.001
Current asthma	5.9	5.7	9.2	<.001	14.9	12.5	16.3	.210	3.5	2.8	6.2	<.001
Symptoms of AR last 12 mo	15.9	15.5	15.2	.804	36.6	34.2	30.1	.095	10.6	7.6	8.8	.020
AR medication last 12 mo	10.4	10.1	9.6	.713	30.5	26.5	23.1	.035	5.2	3.1	3.8	.019
Physician-diagnosed AR	7.3	7.9	5.4	.013	26.4	22.5	15.7	<.001	2.3	1.6	1.1	.028
Current AR	6.4	5.9	4.0	.004	23.4	17.6	11.9	<.001	2.0	0.9	0.6	.003

## Discussion

In Northern Sweden, our 3 cross-sectional studies among 8-year-old children over a 21-year period showed an increase in allergic sensitization to airborne allergens between 1996 and 2006 (from 20.6% to 29.9%) followed by a plateau by 2017. Sensitization to furry animals and pollen was most common in all surveys. The risk factor analyses yielded similar results at all time points, with a family history of allergy as the main risk factor for allergic sensitization, whereas having a cat at home showed consistent negative associations. Several factors implicating exposure to a more diverse microbiome showed negative associations with allergic sensitization, and a dose-response effect by number of such factors was found. The prevalence trends of asthma and AR showed some interesting differences. Although symptoms of the respective diseases remained largely unchanged in prevalence, physician-diagnosed asthma increased but physician-diagnosed AR decreased, and these trends were independent of sensitization to airborne allergens. The association between allergic sensitization and physician-diagnosed asthma weakened over time, whereas its association with AR strengthened.

After decades with increasing prevalence of sensitization to airborne allergens, many studies performed around the millennium shift report a leveling prevalence.^7^^,^^8^^,^^10^^,^^11^ In Northern Sweden, this stabilization in prevalence seems to have occurred somewhat later. The explanation for this finding is not known, but it could be speculated that the level of prevalence of airborne sensitization in the population may play a role. In 1996, 20% of the children in our area were sensitized compared with 30% in 2006. The stabilization of sensitization prevalence in the following decade might reflect a saturation in the population wherein most individuals at risk have developed sensitization. In a population-based study from Greenland, the prevalence increased between 1987 and 1998, and the increase was the largest in the age group with the lowest prevalence in 1987 but less pronounced in the groups with higher prevalence.^9^

The ongoing climate change, resulting in shorter winter seasons in Northern Sweden, may affect the pattern of sensitization. In fact, the vegetation period, defined as the number of days with an average temperature of 5°C, has increased with approximately 8 to 10 days in Northern Sweden during the observation period of 1996 to 2017,^34^ which may have increased the exposure to pollen. If this is the case, then whether it also explains the increase in sensitization to pollen remains an open question and needs further study. An increase in the sensitization to mites, a major sensitizer in warmer areas,^13^^,^^17^ might also be expected. This has, however, not yet been observed in our area of cold and dry climate, with a consistent pattern of pollen and furry animals being the major sources of sensitization. Interestingly, despite the humid climate in Isle of Wight, the high prevalence of mite sensitization decreased among schoolchildren from 2001 to 2012.^13^ Those authors speculated that improvements in housing conditions may have contributed. However, their overall prevalence of airborne sensitization did not change, which is in line with our findings over the most recent decade.

Despite decades of research, we still cannot explain the reason for the increase in allergic sensitization. Apart from genetic susceptibility, often denoted by a family history of allergy,^12^^,^^19^ few other risk factors have been identified. Instead, a widely adopted hypothesis is that a number of lifestyle changes have conferred a lack of immune- stimulating exposures that may be critical for our immune system. Several studies have reported protective effects from factors such as having several siblings,^35^^,^^36^ living at a farm,^21^^,^^22^^,^^24^ living at the countryside,^23^ and having pets at home.^12^^,^^25^, ^26^, ^27^, ^28^ Such factors indicate exposure to a more diverse microbiome,^20^, ^21^, ^22^ and they were also negatively associated with allergic sensitization in our study. Interestingly, a dose-response relationship with the number of such exposures was observed, which supports causality. However, it should be noted that these factors should be seen as proxies in the absence of firm explanatory models on the basis of mechanistic research, and in the present study their prevalence did not decrease, but allergic sensitization increased. Likewise, we were lacking information about factors in early life, such as method of delivery, diet, and use of antibiotics, that have been associated with diversity of the gut microbiome. We chose to include having a cat at home as indicative of increased microbiome diversity although we are well aware that it also has specific effect on sensitization by the production of IgG_4_ antibodies to the cat allergen Fel d 1. This is reported in several studies from the current authors on completely different cohorts,^29^^,^^37^^,^^38^ and in the present study the negative association between cat at home and allergic sensitization was found in all 3 populations. Equally, the adjusted analyses suggest that the effect of cat is stronger than that of dog, which is unlikely to be due to microbiome because it is reported that the level of endotoxin is lower in homes with a cat than in homes with a dog.^39^

An exposure that substantially decreased from 32% in 1996 to 7% in 2017 was maternal smoking. Although related to asthma development,^40^ we did not find any association with allergic sensitization, in line with other studies.^41^^,^^42^ Another interesting aspect is the sex differences wherein boys showed an increased risk for allergic sensitization, although not statistically significant in all surveys. Not all studies report sex differences, but those that do are in line with our results.^42^^,^^43^ The issue regarding infections and allergic sensitization is unclear. Strachan^44^ was one of the first to report a protective effect on atopic diseases by having many siblings. In line with this, it was argued that having many siblings was a marker for infections, implicating a beneficial effect of viral infections against allergic sensitization. However, several later studies have indeed reported that respiratory infections in childhood constitute a risk factor for asthma and wheeze,^45^^,^^46^ particularly among nonallergic children.^12^^,^^47^ Regarding allergic sensitization, the pattern is different. We did not find any association between severe respiratory infections and allergic sensitization in any of the cohorts of the present study, which is in line with several epidemiological studies including detailed analyses of our first cohort.^12^^,^^46^^,^^47^ However, a recent longitudinal study found an association between severe lower respiratory tract infections before 18 months of life and polysensitization at age 5 years.^48^ Because virtually all children have been infected by respiratory viruses within the first 2 years of life, studies among schoolchildren may be affected by recall bias regarding severity, age, and type of early infections.

An unexpected result in our study was the different trends in prevalence of asthma and AR, as presented in Table IV, compared with the trends of allergic sensitization. Because of the strong association between allergic sensitization and asthma and AR, a similar trend would have been expected. The prevalence of asthma increased, however, and most markedly so between 2006 and 2017. Furthermore, the relative and absolute change was larger among nonsensitized children than among sensitized children and occurred during the period when the prevalence of allergic sensitization remained stable.

We have previously reported that the severity of asthma among those with physician-diagnosed asthma has decreased.^49^ Thus, increased awareness in the population and increased diagnostic intensity have probably contributed to the large increase in physician diagnosis and use of asthma medication, which was seen parallel to a stable prevalence of wheeze. To some extent, the lack of increased symptom prevalence may be explained by increased use and improvements in asthma treatment, but also by the observed decrease in secondhand smoke from parents, which is a known risk factor for asthma symptoms.^50^ Our data are in line with worldwide data for children also showing a stable prevalence of current wheeze and a decreasing prevalence of severe asthma symptoms.^2^ However, because the focus of our article is allergic sensitization, the trends in asthma will require further exploration in separate studies.

Regarding AR, the decreasing prevalence of symptoms and diagnosis may be due to increased availability of effective treatments. In contrast to treatment of asthma, effective medications for AR are now readily available at pharmacies over the counter, and prescription drugs are not recommended as first line of treatment. Thus, fewer symptoms and lower prevalence of physician-diagnosed AR may in part be explained by differences in management over time. The proportion of children with physician diagnosis of AR that could be attributed to allergic sensitization became larger by time, whereas the opposite was found for asthma. Globally, and in line with our results, the prevalence of AR does not seem to increase any more even if the results vary substantially both between and within countries.^3^ Taken together, our results are in line with the recent large multicenter studies showing diverging trends of asthma and AR.^2^^,^^3^

Key strengths of our study are the identical study design regarding age, area, and methods used in the 3 cross-sectional studies, and the exceptionally high response rates in all surveys, which allowed us to estimate population prevalence with a high validity. The consistency of our findings regarding prevalence trends over time is also supported by the sensitivity analyses based on higher cutoff values for a positive SPT response showing similar patterns. The SPT responses have been validated by analyses of specific IgE both at age 8 years and at age 12 years in the first 2 cohorts with good agreement.^31^ A limitation is that no such validation was performed in the third cohort. However, the SPTs followed the same protocol in all surveys and the tests were performed by a small number of mainly the same personnel. Another limitation is that we do not have data regarding sensitization in early age because our cohorts were recruited at age 7 to 8 years, and young age at first sensitization is an important factor regarding the probability of developing clinical allergy.^16^^,^^51^ Thus, we do not know whether age at first sensitization has changed in the cohorts and in that way have contributed to the different trends in sensitization, asthma, and AR. Nevertheless, the observed high prevalence of allergic sensitization may still contribute to future increases in asthma and AR during later childhood, adolescence, or young adulthood.^5^^,^^16^

We found different trends of allergic sensitization, asthma, and AR over the 21-year observation period from 1996 to 2017. Allergic sensitization increased from 21% in 1996 to 30% in 2006, and remained at the same level in 2017. Having a cat at home as well as several factors suggestive of exposure to a more diverse microbiome showed a negative association with allergic sensitization, and a dose-response effect by number of such factors was found. The prevalence of physician-diagnosed asthma increased significantly among both sensitized and nonsensitized children, whereas the prevalence of AR decreased. These diverging trends resulted in a weakened association between sensitization and asthma, whereas the association with AR was strengthened. Data on symptoms and medication use implicate changes in the management of allergic diseases over time, which warrant further investigation.

## Disclosure statement

The study was funded by grants from the 10.13039/501100003793Swedish Heart-Lung Foundation, the Swedish Asthma-Allergy Foundation, the Swedish Research Council, the Swedish Foundation for Health Care Science and Allergy Research (Vårdal), Norrbotten County Council, Visare Norr, and ALF (a regional agreement between Umeå University and Västerbotten County Council). Additional support was provided by ALK-Abelló (Hørsholm, Denmark).

Disclosure of potential conflict of interest: The authors declare that they have no relevant conflicts of interest.

**Key messages**

• After a substantial increase in allergic sensitization among children between 1996 and 2006, the prevalence plateaued by 2017.
• Risk factor patterns for allergic sensitization were consistent over time.
• The associations between allergic sensitization, asthma, and AR changed over the 21-year study period, with asthma becoming less strongly associated with sensitization and AR becoming more strongly associated.
