# Supplementary material for: Prevalence and risk factors for allergic sensitization: 3 cross-sectional studies among schoolchildren from 1996 to 2017
Source: J Allergy Clin Immunol Glob. 2023 Jul 19;2(4):100150. doi: 10.1016/j.jacig.2023.100150 (PMC10510014; doi:10.1016/j.jacig.2023.100150)
Supplement: Supplementary Tables [file mmc1.docx]

Online Repository Table 1. Definitions of asthma, allergic rhinitis (AR) and potential risk factors.

| **Definition** | **Question in the questionnaire completed by the parents.** |
| --- | --- |
| **Asthma and allergic rhinitis** | |
| Physician diagnosed asthma | Has your child been diagnosed by a physician as having asthma? |
| Wheeze last 12 months | Has your child had wheezing or whistling in the chest in the last 12 months? |
| Asthma medication last 12 months | How often has your child had to use asthma medication in the last 12 months (sometimes, often/periodically, or everyday)? |
| Current asthma | Physician diagnosed asthma and either wheeze or use of asthma medication during the last 12 months. |
| Physician diagnosed allergic rhinitis (AR) | Has your child been diagnosed by a physician as having hay fever or allergic nose/eye problem? |
| Symptoms of AR last 12 months | In the last 12 months, has your child had a problem with sneezing, or a runny, or a blocked nose when he/she did not have a cold? |
| AR medication last 12 months | How often has your child had to use medication for allergic nose/eye problems in the last 12 months (sometimes, often/periodically, or everyday)? |
| Current allergic rhinitis | Physician diagnosed allergic rhinitis and either symptoms of AR or use of AR medication during the last 12 months. |
| **Potential risk factors** | |
| Family history allergy | Are there allergic nose or eye problems in mother or father? |
| Number of siblings | How many siblings does your child have? |
| Type of living 1^st^ year of life | Type of setting first year of life? (house or apartment) |
| Ever cat at home | Do You presently keep or have You previously kept cat at home during the time your child was growing up? |
| Ever dog at home | Do You presently keep or have You previously kept dog at home during the time your child was growing up? |
| Furry animal at home first two years of life | Where there any furred animals in the home at any time period during your child’s first two years of life? |
| Rural living first year of life | Type of setting first year of life? (urban or rural) |
| Ever living on a farm | During the time your child was growing up: Do you currently have or have you previously had a farm? |
| Birthweight <2500 grams | What was your child’s birth weight? |
| Breastfeeding <3 months | Until what age was your child fed breast milk? |
| Any severe respiratory infection | Has your child had whooping cough, croup, pneumonia or severe airway disease, e.g. RS Virus? |
| Maternal smoking | Present smoking habits within the family. Please tick the applicable boxes in the table. Mother smokes > 0-4 cigarette per day. |
| Maternal smoking in pregnancy | Did the child’s mother smoke during the pregnancy? |
| Dampness at home | Signs of damage from dampness or moulds? (yes to current or previous residence)? |
| Heavy traffic road close to home | A large busy road or a frequented bus stop within 200 meters of the home? (Yes to current or previous residence) |

| Online repository Table 2. Prevalence (%) of allergic sensitization based on different cut-off for a positive skin prick test (SPT), by study year. | | | | | | | | | | | | | | |
| --- | --- | --- | --- | --- | --- | --- | --- | --- | --- | --- | --- | --- | --- | --- |
|  |  |  |  |  |  |  |  |  |  |  |  |  |  |  |
|  | Positive response defined as ≥ 3mm | | | |  | Positive response defined as ≥5 mm | | | |  | Positive response defined as ≥ 7mm | | | |
| Allergen | 1996 | 2006 | 2017 |  |  | 1996 | 2006 | 2017 |  |  | 1996 | 2006 | 2017 |  |
| Any positive SPT* | 20,6 | 29,9 | 30 | <0.001 |  | 13,5 | 19,6 | 21,1 | <0.001 |  | 7,7 | 10,2 | 11,5 | <0.001 |
|  |  |  |  |  |  |  |  |  |  |  |  |  |  |  |
| Birch | 7.9 | 13.1 | 11.7 | <0.001 |  | 4.0 | 5.9 | 7.5 | <0.001 |  | 1.4 | 1.4 | 2.4 | 0.026 |
| Timothy | 6.7 | 10.9 | 12.5 | <0.001 |  | 3.0 | 5.4 | 7.0 | <0.001 |  | 1.3 | 1.7 | 2.5 | 0.018 |
| Cat | 13.4 | 19.1 | 17.4 | <0.001 |  | 8.8 | 12.8 | 12.7 | <0.001 |  | 4.2 | 5.6 | 6.6 | 0.003 |
| Dog | 8.7 | 15.7 | 14.8 | <0.001 |  | 3.4 | 6.0 | 6.4 | <0.001 |  | 1.3 | 2.3 | 1.9 | 0.061 |
| Horse | 6.1 | 9.0 | 7.0 | 0.002 |  | 4.1 | 6.3 | 6.0 | 0.005 |  | 2.3 | 3.6 | 3.2 | 0.055 |
| *Defined as a positive response to any of birch, timothy, mugwort, cat, dog, horse, Der pteronyssinus, Der Farinae, Cladosporium or Alternaria. | | | | | | | | | | | | | | |

Online Repository Table 3 The mean size, mm (standard deviation) of the reactions for the most common allergens in 1996, 2006 and 2017.

|  | 1996 | 2006 | 2017 |
| --- | --- | --- | --- |
| Birch, mm(SD) | 4.7 (1.7) | 4.5 (1.6) | 5.1 (1.8) |
| Timothy, mm(SD) | 4.7 (2.1) | 4.8 (2.1) | 4.9 (1.9) |
| Dog, mm(SD) | 4.4 (2.0) | 4.5 (1.9) | 4.5 (1.9) |
| Cat, mm(SD) | 5.6 (2.3) | 5.6 (2.3) | 5.9 (2.3) |
|  |  |  |  |
|  |  |  |  |
